# Reconstitution and Coupling of DNA Replication and Segregation in a Biomimetic System

**DOI:** 10.1002/cbic.201900299

**Published:** 2019-08-28

**Authors:** Daniel Hürtgen, Judita Mascarenhas, Michael Heymann, Seán M. Murray, Petra Schwille, Victor Sourjik

**Affiliations:** ^1^ Max Planck Institute for Terrestrial Microbiology & LOEWE Center for Synthetic Microbiology (Synmikro) Karl-von-Frisch Strasse 16 35043 Marburg Germany; ^2^ Max Planck Institute of Biochemistry Am Klopferspitz 18 82152 Martinsried Germany

**Keywords:** DNA nanoparticles, DNA replication, DNA segregation, minimal cell, ParM, T7

## Abstract

A biomimetic system capable of replication and segregation of genetic material constitutes an essential component for the future design of a minimal synthetic cell. Here we have used the simple T7 bacteriophage system and the plasmid‐derived ParMRC system to establish in vitro DNA replication and DNA segregation, respectively. These processes were incorporated into biomimetic compartments providing an enclosed reaction space. The functional lifetime of the encapsulated segregation system could be prolonged by equipping it with ATP‐regenerating and oxygen‐scavenging systems. Finally, we showed that DNA replication and segregation processes could be coupled in vitro by using condensed DNA nanoparticles resulting from DNA replication. ParM spindles extended over tens of micrometers and could thus be used for segregation in compartments that are significantly longer than bacterial cell size. Overall, this work demonstrates the successful bottom‐up assembly and coupling of molecular machines that mediate replication and segregation, thus providing an important step towards the development of a fully functional minimal cell.

## Graphical abstract

**Minimal cells**: Replication and segregation of genetic material constitutes an essential feature for the design of a minimal synthetic cell. Here we have used the simple T7 bacteriophage system and the plasmid‐derived ParMRC segregation system to establish these processes in vitro. We further showed that DNA replication and DNA segregation could be coupled by using condensed DNA nanoparticles.

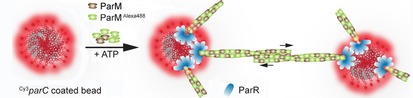

## Introduction

The creation of cell‐mimicking systems relies on combining a minimal set of functionalities that are inspired by essential cellular features and are either directly derived from biological systems or reconstituted by using unnatural parts.^1^, ^2^ The capabilities to replicate and subsequently to segregate replicated genetic information are among these essential features.

DNA replication systems of various degrees of complexity have been successfully reconstituted.^3^, ^4^, ^5^, ^6^, ^7^, ^8^ In particular, mesophilic bacteriophages offer attractive choices when developing a replication apparatus for a minimal cell, due to their robustness and the small set of components required. Polymerases from bacteriophages T4, T7, and Phi29 are commonly used as tools in molecular biology^9^, ^10^ and can generate high DNA yields based on multi‐primed rolling‐circle amplification (RCA).^11^ Recently, de novo synthesis and assembly of functional DNA replication components by using an in vitro transcription and translation system has been demonstrated in the case of the Phi29 bacteriophage. Phi29‐mediated RCA synthesizes long concatamers of linear double‐stranded DNA, which could be recircularized through Cre‐lox recombination.^12^ The T7 replisome is another well understood system suitable for highly processive RCA of DNA. It requires four core proteins, including DNA polymerase Gp5, which complexes with *Escherichia coli* host protein thioredoxin, the bifunctional helicase‐primase Gp4, and single‐stranded DNA‐binding protein Gp2.5.^13^

To minimize the risk of loss of genetic information during cell division, cells further possess DNA segregation machineries. Eukaryotes use dynamic spindles consisting of microtubules that rapidly reorganize by means of polymerization and depolymerization and serve as tracks for molecular motors for chromosome transport.^14^, ^15^, ^16^ In contrast, prokaryotes have evolved a more diverse range of DNA segregation mechanisms,^17^, ^18^ in which Walker‐A ATPases of the ParA‐type commonly partition chromosomal origins of replication.^19^, ^20^, ^21^, ^22^, ^23^, ^24^, ^25^, ^26^, ^27^

Furthermore, extrachromosomal plasmids possess their own filament‐based DNA segregating systems, typically containing three elements: a centromeric DNA sequence, a DNA‐binding protein, and an ATPase.^28^, ^29^, ^30^, ^31^ The most prominent classes of these partitioning systems are type I segregation systems employing ParA‐type ATPases (similar to the chromosome‐segregating systems mentioned above), and type II segregation systems employing actin‐like ATPases of the ParM type.^28^ The dynamically unstable ParM polymers consist of polar, left‐handed, double‐helical filaments and are tethered to plasmid DNA at the growing (+) end through a helical accessory protein complex.^32^ The ParMRC system of the low‐copy‐number plasmid R1,^33^ which as well as ParM also includes the centromeric *parC* site and the *parC*‐binding protein ParR, is the most thoroughly characterized prokaryotic segregation machinery^34^, ^35^ that has also been reconstituted in vitro.^36^

Finally, compartmentalization of cellular functions is a hallmark of all living systems,^37^ and several cellular processes have been successfully reconstituted in biomimetic compartments.^38^, ^39^, ^40^, ^41^ In this work, we reconstituted the core functional aspects of replication and segregation, using the T7 system to replicate circular plasmids and the Cre‐lox system to regenerate them, as well as the ParMRC system for DNA segregation. Furthermore, we showed that DNA replication could be coupled to the segregation process by using condensed DNA nanoparticles. These minimal replisome and segrosome machineries were further incorporated into biomimetic compartments.

## Results

### Minimal replisome reconstitution

**Figure 1:**
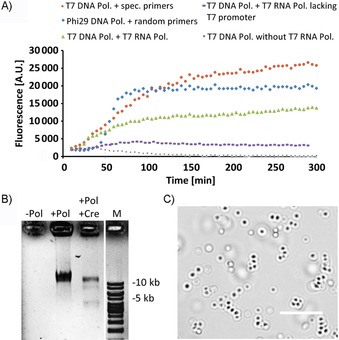
Replication, recircularization, and compaction of a plasmid containing the T7 promoter. A) Time course of RCA‐based replication of pRepC plasmid containing the T7 promoter and *loxP* sites (depicted in Figure S1), measured as fluorescence of the DNA‐binding PicoGreen dye. Reaction mixtures contained, as indicated, Phi29 DNA polymerase, T7 DNA polymerase, and T7 RNA polymerase, as well as specific or random primers. For a control reaction with T7 DNA polymerase and T7 RNA polymerase, a pQE30 plasmid lacking the T7 promoter was used. B) Recircularization of the replicated plasmid, mediated by Cre recombinase. Where indicated, Cre recombinase was added after 16 h of replication, and the reaction mixture was incubated for another 30 min. Reaction mixtures were separated along with a DNA ladder (1 kb) on a Midori‐green stained agarose gel. The lower band migrating below 5 kb corresponds to the circularized DNA, whereas larger products apparently correspond to linear concatamers (Figure S2). C) DNA nanoparticles emerging upon prolonged (>12 h) T7 DNA replication reaction. Scale bar: 10 μm.

To reconstitute the isothermal RCA replication of circular plasmids, we first used the complete T7 replication system, consisting of four proteins: T7 DNA polymerase lacking the exonuclease activity,^42^ thioredoxin, helicase Gp4, and the ssDNA‐binding protein Gp2.5. The template plasmid pRepC contained the promoter for T7 RNA polymerase, a *parC* locus, and a *loxP* site, to enable transcription, segregation, and recircularizationrespectively. We observed robust DNA replication through the action of this T7 in vitro system in the presence of specific primers that anneal on both strands of the double‐stranded template plasmid (Figure 1 A and Figure S1 in the Supporting Information). The observed reaction kinetics were comparable with those of replication mediated by the Phi29 DNA polymerase in the presence of random primers.

Surprisingly, DNA amplification through the action of T7 polymerase in the presence of sequence‐specific primers could be observed even in the absence of the accessory proteins, consistent with previous reports of the T7 polymerase strand displacement activity.^42^, ^43^ In the presence of T7 RNA polymerase, DNA amplification through the action of the complete T7 system also proceeded without external primers, thus suggesting that the RNA polymerase activity is sufficient to initiate DNA replication. Consistently with this, no RNA‐polymerase‐primed replication was observed in case of the template plasmid lacking the T7 promoter. Finally, circular plasmids could be regenerated from the linear RCA concatamers by recombination/circularization of DNA using Cre recombinase. This enzyme recognizes the *loxP* sites on the plasmid, resulting in at least partial regeneration of the circular plasmid (Figure 1 B). To avoid possible interference between recombination and replication,^12^ Cre recombinase was only added after the plasmid replication had been allowed to proceed for 16 h. Recircularization of DNA was confirmed by subsequent digest of the recombination products with an exonuclease (Figure S2) that enzymatically removes linear fragments.^44^

We further observed, upon prolonged replication (6–12 h), the emergence of spherical particles of <3 μm in diameter in the reaction mixture (Figure 1 C). As reported before, these nanoparticles are co‐precipitates of DNA and magnesium pyrophosphate that accumulates as the byproduct of DNA synthesis in the absence of pyrophosphate kinase.^45^ Consistent with this, the observed nanoparticles could be stained by DNA‐binding dye (Figure S3). Notably, previous work had shown that the nanoparticles are still accessible to transcription and translation machineries.^46^, ^47^

## Minimal segrosome reconstitution

**Figure 2:**
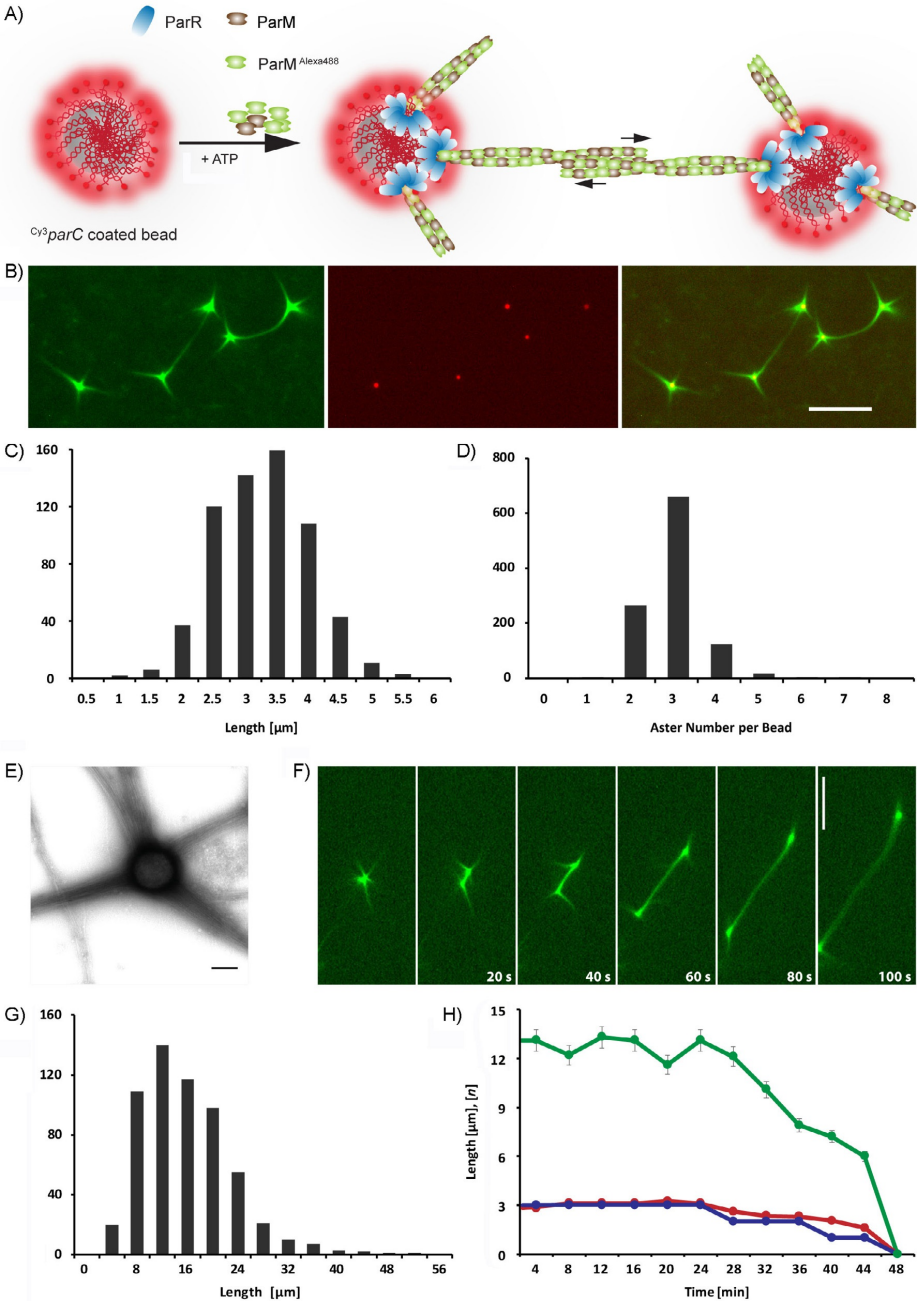
DNA segregation by the ParMRC system. A) Schematic representation of in vitro segregation of Cy3‐labeled *parC* sequences (red) bound to beads through streptavidin/biotin chemistry. Polymerization of Alexa488‐labeled ParM (green) leads to filament formation and segregation of beads in dependence on ParR (blue). B) In vitro reconstitution of the ParMRC system, with ParM (green) forming spindles connecting two beads, as well as free asters that are linked to the *parC*‐coated beads (red) by ParR (unlabeled). Reaction mixtures containing 5 μm ParM, 250 nm ParR, and 14 pm *parC*‐coated beads were mixed with 10 mm ATP to induce polymerization. Scale bar: 5 μm. C) Distribution of aster length, and D) number of asters per bead. E) TEM image of ParM filament bundles growing from a bead. Scale bar: 200 nm. F) Time‐lapse series of an elongating ParM spindle pushing *parC*‐bound beads apart. Scale bar: 10 μm. G) Distributions of spindle lengths. H) Stability of spindle length (green), aster length (red), and asters per bead (blue) over time.

To implement faithful DNA segregation in vitro, we reconstituted the R1‐ParMRC segregation system. For visualization of the segregation process, we adapted the microbead approach of Garner et al.,^36^ in which biotinylated Cy3‐labeled *parC* DNA fragments serving as ParR anchors were bound to 300–350 nm streptavidin‐coated beads (Figure 2 A). We observed that upon mixing with ParR and ATP, asters and spindles of Alexa488‐labeled ParM were formed at the beads (Figure 2 B, Movies S1 and S2). Here, the term “aster” refers to ParM filaments that are attached to the ParRC complex at one end only, seen as filaments growing up to ≈3 μm (Figure 2 C). On average, about three asters per bead were formed (Figure 2 D). Transmission electron microscopy (TEM) showed that these are bundles of multiple (>20) filaments growing from the microbead surface (Figure 2 E). In contrast, the term “spindle” refers to ParM filaments that are attached in a bipolar manner to the ParRC complexes, resulting in bead segregation (Figure 2 F). These attached ParM spindles are much longer than asters, with a median length of 12.9 μm (Figure 2 G). Both spindles and asters remain stable under our conditions up to 30 min (Figure 2 H). We further observed multipolar spindles interconnecting more than two beads (Figure S4 and Movie S2).

## DNA segregation as a dynamic event

**Figure 3:**
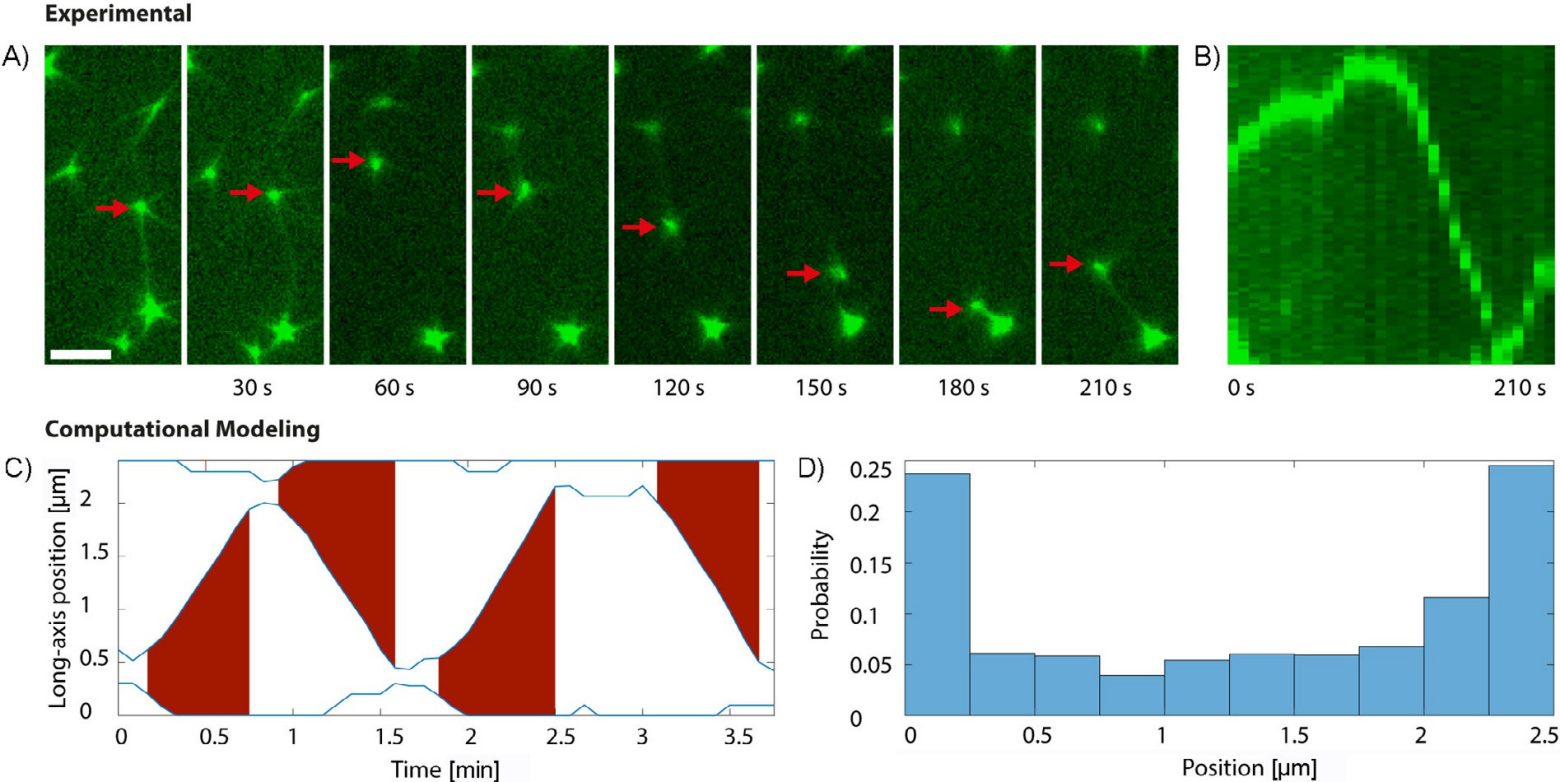
DNA segregation as an ongoing, dynamic event. A) Time‐lapse imaging of one *parC*‐covered bead (red arrow) undergoing the segregation process multiple times between two neighboring beads within 210 s. Scale bar: 5 μm. B) A kymograph shows the localization dynamics of this bead. C) Simulation of ParMRC‐mediated bead segregation. Shown are the positions of three beads along the long axis of a rectangular compartment (blue lines). Red areas indicate spindle formation and extension. D) Distribution of bead positions along the long axis of the compartment resulting from simulation in (C), with approximately equal number of beads being positioned at each end of the compartment.

During segregation of artificial beads, we observed relay behavior in which a bead was moved back and forth between two or more other beads (Figure 3 A, B, Movies S3 and S4). This is reminiscent of the dynamics of ParMRC‐segregated plasmids in vivo,^34^ in which plasmids are rapidly (within ≈30 s) and frequently shuttled from one pole of the cell to the other. To better understand the potential role of these dynamics within biomimetic compartments, we developed stochastic simulations of our ParMRC bead system within a confined compartment (Figure 3 C, D). Here, simulated beads move either by diffusion, by aster growth pushing them away from a boundary, or by forming a spindle with another bead. Spindles that can grow no further break. When simulated within a narrow rectangular compartment (with a 4:1 aspect ratio), we found that beads were shuttled back and forth between the compartment ends, similarly to what was observed experimentally (Figure 3 C). Interestingly, we found that these repeated segregation events led to robust bead partitioning: that is, equal numbers of beads being positioned at opposite ends of the compartment (Figure 3 D). Because spindles are more likely to form at the compartment end with the most beads, on average a bead is moved from that end to the other. The continuous repetition of this process leads, on average, to an equal number of beads at each end of the compartment, with relatively little variation. Thus, the observed dynamics would be expected to increase the efficiency of DNA segregation.

## DNA segregation in microcompartments

**Figure 4:**
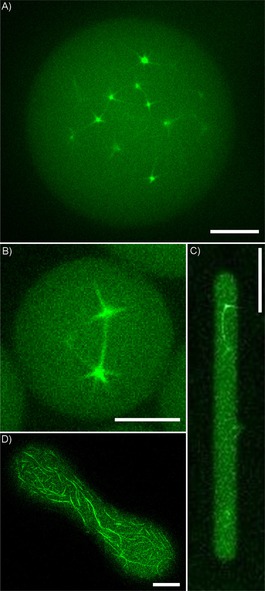
Segregation of *parC*‐coated beads in microcompartments. Several different types of biomimetic confinement were tested. A), B) Water‐in‐oil droplets. Scale bars: 10 μm and 5 μm, respectively. C) Water‐in‐oil droplet squeezed into PDMS channel. Scale bar: 20 μm. D) Half‐open PDMS channel covered with lipid bilayer isolated from *E*. *coli*. Scale bar: 10 μm.

To demonstrate segregation under spatial confinement experimentally, we encapsulated the segregation machinery in several different types of microconfinement (Figure 4). These included water‐in‐oil emulsion droplets (Figure 4 A, B), water‐in‐oil droplets squeezed into a microfluidic PDMS channel (Figure 4 C), and half‐open PDMS channels sealed with *E*. *coli* lipids (Figure 4 D), as well as BSA‐coated microfluidic PDMS channels (Movie S5). All tested confinements sustained dynamic spindle formation and hence bead segregation, with no apparent interference with the ParMRC system, although no segregating spindles could be observed in water‐in‐oil droplets with diameters below a threshold of approx. 10 μm, most likely due to a limiting number of molecules. Notably, ParM polymers aligned with the long axes of the tested compartments, thus indicating that the growing spindles can orient themselves by exerting a force to slide against the walls of the compartment, although no deformation of the water‐in‐oil droplets could be observed.

## Lifetime extension of segregation in microcompartments by ATP‐regenerating and oxygen‐scavenging systems

**Figure 5:**
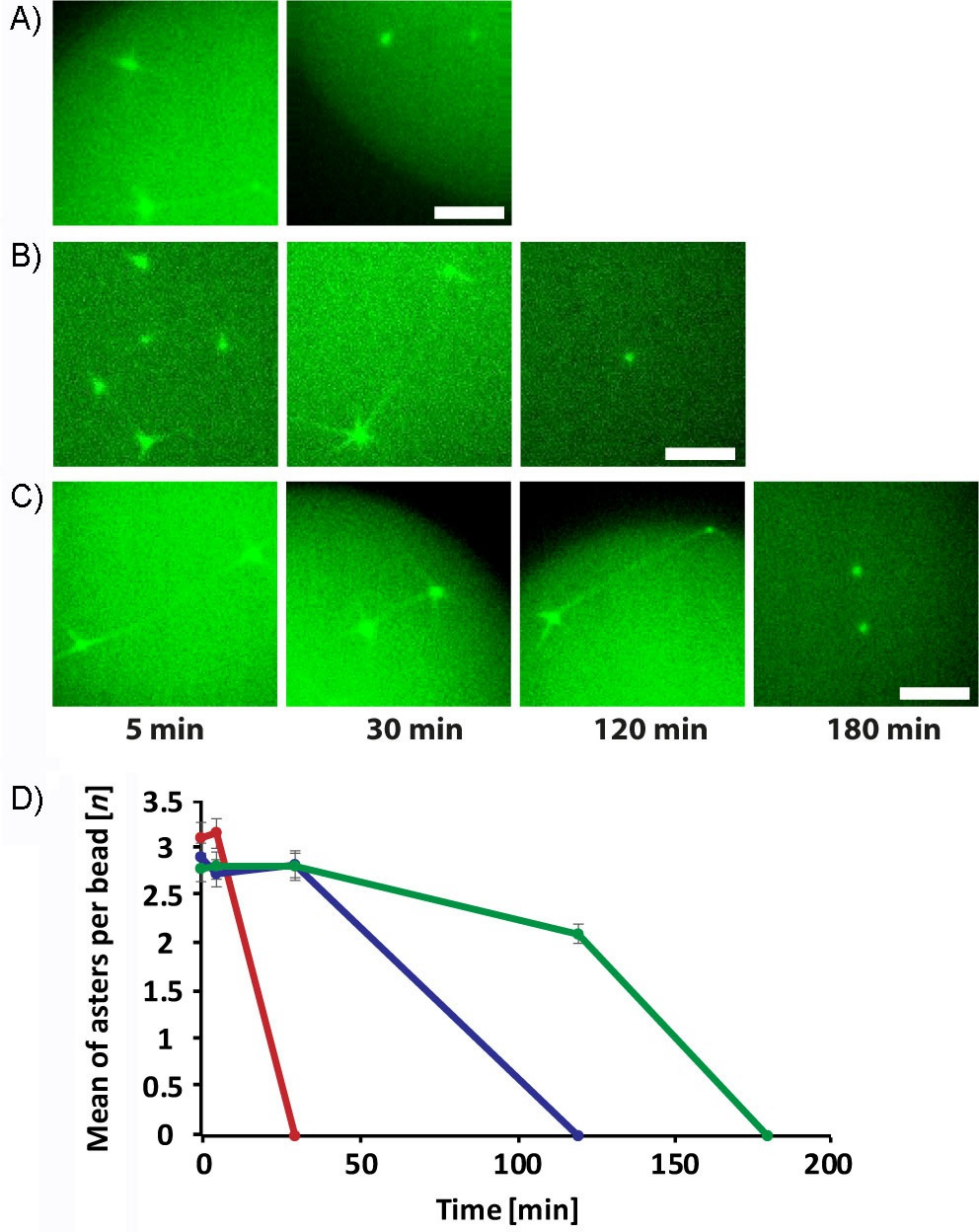
Lifetime extension of the segregation system in water‐in‐oil droplets. Formation of ParM spindles at indicated time points for the system reconstituted A) as in Figure 2, B) with ATP regeneration, and C) with both ATP regeneration and an oxygen‐scavenger system. Scale bars: 5 μm. D) Quantification of aster formation in experiments shown in A (•), B (•) and C (•). Error bars indicate standard deviations.

Active segregation by dynamic ParM filaments relies on energy consumption, such that ATP might quickly become a limiting factor, especially under spatial confinement conditions. Indeed, both in the open (Figure 2 H) and in the confined (Figure 5 A) systems, ParM spindles were no longer observed after 30 to 50 min of incubation. The lifetime of the segregation system could indeed be significantly extended by including an ATP regeneration system, based on phosphocreatine and creatine kinase (Figures 5 B and S5). Further extension could be achieved by supplementing the reaction mixture with an enzymatic oxygen scavenger based on pyranose oxidase and catalase,^48^ to prevent oxidative damage of the proteins and especially the ParM filaments (Figure 5 C, D).

## Segregation of DNA nanoparticles

**Figure 6:**
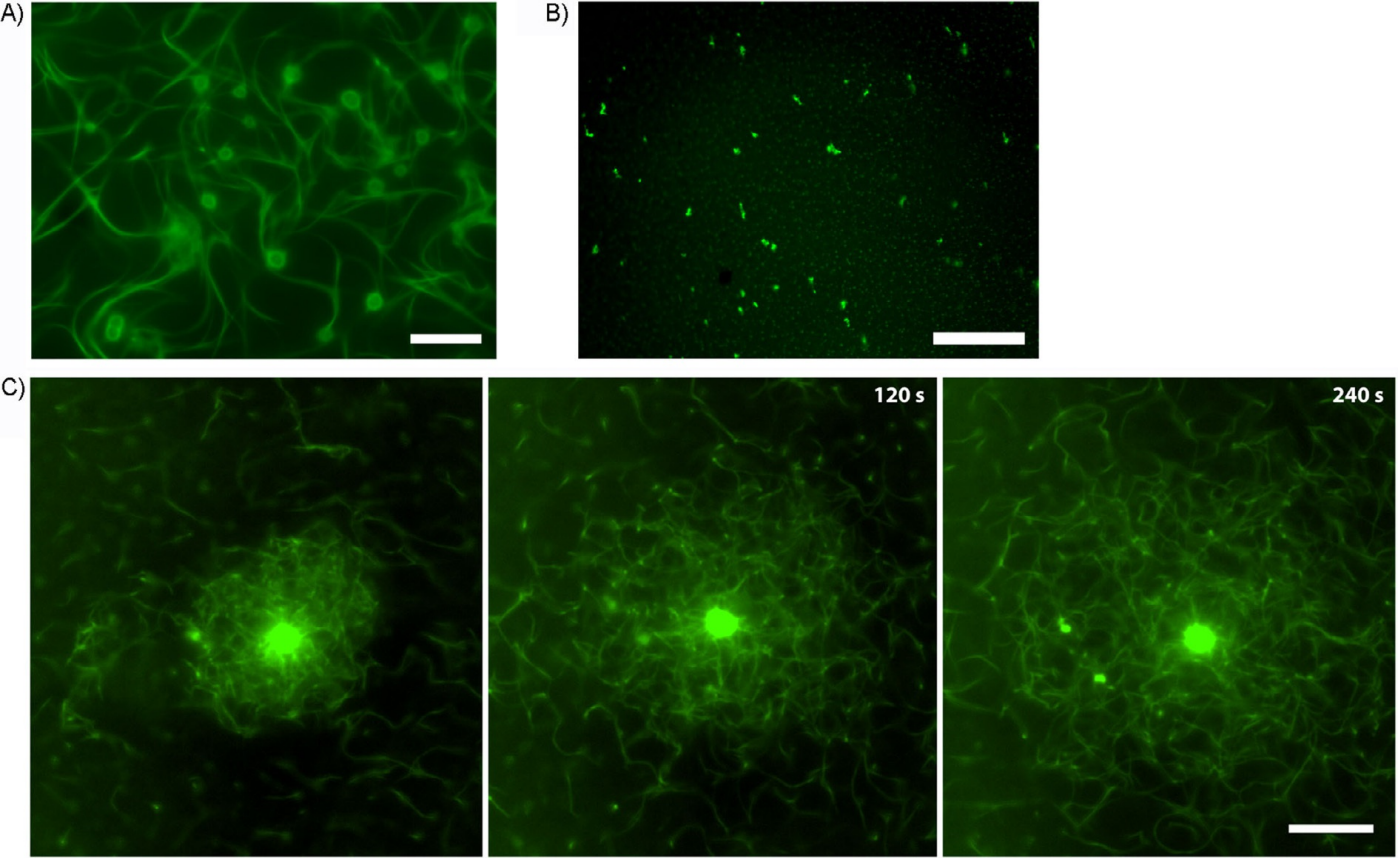
Segregation of DNA nanoparticles by the ParMR system. A) DNA nanoparticles formed upon replication of the *parC*‐containing pRepC plasmid associate with Alexa488‐labeled ParM in a ParR‐dependent manner. Reaction conditions were as in Figure 2 F. B) Control reaction with pUC19 plasmid lacking *parC*. C) Time series of dynamic ParM filament meshwork formed around one large DNA nanoparticle that shears off and pushes apart smaller nanoparticles. Scale bars: 20 μm.

We next explored whether DNA nanoparticles formed during prolonged replication (Figure 1 C) could be segregated by ParM spindles. Upon addition of isolated DNA nanoparticles formed by the *parC*‐containing plasmid to ParMR, ParM binding and filament formation on these nanoparticles were observed (Figure 6 A). In contrast, control nanoparticles lacking *parC* sites did not induce ParM filament formation (Figure 6 B), thus confirming the specificity of the reaction and additionally showing that *parC* DNA is accessible within the nanoparticles. ParM filaments grew radially from the *parC*‐containing nanoparticles (Figure 6 A, C and Movies S6–S8) and formed stable bundles connecting nanoparticles, similar to the bipolar structures observed when using artificial beads. These ParM‐connected nanoparticles with multiple *parC* sites formed a dense dynamic meshwork that could push and disperse groups of nanoparticles (Figure 6 C and Movies S6–S8).

## Discussion

The design of a minimal replication/segregation machinery constitutes one of the major challenges on the pathway towards the creation of a minimal synthetic cell. In this work, we successfully reconstituted modules for DNA replication and DNA segregation by using bacteriophage and plasmid systems, encapsulated these reactions in microcompartments, and showed that they could be coupled through the formation of DNA nanoparticles.

Here we used RCA replication mediated by T7 DNA polymerase, in which replication could be initiated in a primer‐free system by T7 RNA polymerase,^49^ thus providing an attractive alternative for minimal self‐replication systems. The linear replication products resulting from RCA could be at least partly resolved by using Cre recombinase, in an approach similar to that recently proposed for the Phi29 replication system.^12^ The efficiency of template recircularization and its coupling to replication could be further improved by optimizing the enzyme/template ratio and by using a Cre recombinase mutant that does not interfere with the DNA replication reaction.^12^

Out of the different cytomotive filament‐based systems of bacterial plasmid segregation,^18^, ^30^, ^31^, ^50^ we used the best‐studied R1/ParMRC machinery.^50^ DNA segregation by this system was reconstituted by using *parC*‐coated beads that, similar to a previous study,^36^ induced ParR‐dependent nucleation of ParM filaments. Multiple filaments further bundled into asters, as well as into spindles connecting the beads. Whereas asters were only a few micrometers in length, spindles extended over tens of micrometers and could thus be used for segregation of comparatively large DNA units (comparable in size to 300–350 nm beads) in compartments that were significantly longer than bacterial cell size.

Further, the observed oscillatory dynamics of the ParMRC segregation machinery are interesting in the context of its in vivo function. Although type II plasmid segregation is typically assumed to be a unique event that occurs prior to cell division,^32^, ^51^, ^52^ our observations suggest that it might rather be a dynamic, extended process. This conclusion is supported by the computational model of spatiotemporal segregation, and it can explain previously observed plasmid dynamics.^34^

Because any future design of a minimal cell‐like system must likely involve compartmentalization, we also verified the functionality of the segregation system in microcompartments, as has previously been done for several protein machineries including actin,^53^, ^54^ the Min system,^40^, ^55^ and MreB.^56^, ^57^ Although the compartmentalization itself apparently did not interfere with the function of the ParMRC system, the performance of the encapsulated system deteriorated more rapidly than in the case of the reactions performed in bulk. This was likely due to ATP depletion and/or the ADP poisoning effect^32^, ^58^, ^59^ leading to depolymerization of ParM.^32^ The lifetime of the system could be prolonged by including an ATP‐regenerating system that counteracts both the ATP depletion and ADP accumulation. Yet a further increase in performance duration resulted from incorporation of an oxygen‐scavenging system based on pyranose oxidase and catalase.^48^

With regard to the application of the ParMRC system in the cell‐free segregation systems, we observed preferential alignment of ParM filaments along the long axis of the confinement. However, at least in water‐in‐oil droplets, ParM filaments did not exert sufficient force to cause compartment deformation, unlike actin/myosin‐based filaments in membrane vesicles.^60^ However, this is not surprising, in view of the high surface tension of droplets compared to vesicles. Application of the ParMRC system for in vitro segregation should be largely facilitated by the condensation of replicated DNA. In a bacterial cell, DNA is condensed both by supercoiling and by several DNA‐binding proteins. Although in vitro reconstitution of these activities might in principle be possible, the inorganic DNA precipitates produced as by‐products of in vitro DNA synthesis^46^, ^47^ provide a promising alternative. As these DNA nanoparticles are accessible for protein binding and functional as templates for transcription,^47^ they might indeed mimic the condensed state of cellular DNA and serve as templates for successive replication in minimal systems. Moreover, DNA nanoparticles might be superior to individual plasmids as segregation substrates, as the presence of multiple *parC* sites and slow diffusion facilitate nucleation of multifilamented bipolar spindles.

One current limitation of DNA nanoparticles is their heterogeneous size, which has a major impact on their segregation. We observed that only nanoparticles of <1 μm in diameter could be efficiently segregated, whereas the presence of small nanoparticles led to the emergence of multiple nucleation sites and thus of a filament meshwork instead of the welldefined bipolar spindles. Nevertheless, more uniform sizes of DNA nanoparticles might be achievable through restriction of reaction volumes, as well as by optimization of replication conditions.

In summary, although a number of challenges, such as optimization and synchronization of all processes remain, we were able to use bacterial phage and plasmid proteins to reconstitute DNA replication and segregation, the two key features of minimal cell design. These enzymatic systems were functional in different microcompartments, and the segregation process could be sustained over extended periods of time with the help of energy regeneration. We further showed that replication and segregation of plasmid DNA can be connected by using DNA nanoparticles, and that site‐specific recombination can be used to resolve plasmid concatamers emerging from the RCA replication. Finally, the observed oscillatory dynamics of the ParM‐mediated segregation are likely important for the function of this system in vivo.

## Experimental Section

**Strains**: *E*. *coli* DH5a strain was used for cloning purposes. Strains BL21(DE3) or M15 with pREP4 were used for protein production.

For protein expression, encoding sequences of T7 helicase Gp4 and single‐stranded DNA‐binding protein Gp2.5 were amplified by PCR from the T7 DNA (Bioron) and cloned in frame at BamHI and HindIII sites of pQE30 (Qiagen).

**In vitro replication**: The pRepC plasmid used for in vitro replication was derived from a pET11a plasmid containing a T7 promoter, a *parC* site, and a 34 bp Cre‐lox site. For T7 DNA replication, 0.1 ng μL^−1^ of template plasmid was used. The reaction mixture consisted of the thioredoxin‐reconstituted (Sigma) T7^exo−^ DNA polymerase (80 nm, a kind gift from Smita S. Patel, Rutgers School of Public Health, NJ), T7 helicase unit Gp4 (60 nm), ssDNA‐binding protein Gp2.5 (4 μm), dTTP (5 mm), and dNTPs (1.25 mm). To provide continuous reverse‐stranded synthesis, external specific primers (0.4 μm) that primed adjacently on opposite strands were used. Reaction components were mixed in a replication buffer [Tris**⋅**HCl (pH 7.5, 40 mm), potassium glutamate (50 mm), MgCl_2_ (10 mm), EDTA (2 mm), dithiothreitol (DTT, 1 mm), PEG 4000 (1 %)]. For self‐primed DNA replication, T7 RNA polymerase (NEB, 1 U) and NTPs (0.2 mm) were added to stimulate the strand opening and RNA priming at the T7 promoter region on the replication template plasmid. For Phi29 DNA replication, Phi29 DNA polymerase (NEB, 1 U) was used with random primers (2.5 μm) and dNTPs (1.25 mm). The replication reactions were monitored for 4–12 h at 30 °C. The replication kinetics were measured with a plate reader (Tecan) and use of DNA‐binding PicoGreen dye in the reaction. To circularize the replicated DNA, Cre recombinase (NEB, 1 U) was added to the 1:10 diluted replication reaction mixture in 20 μL reaction volume and further incubated for 30 min. To confirm the emergence of recircularized plasmid upon Cre recombination, the reaction mixture was treated for 2 h with exonuclease V (NEB, 50 mU μL^−1^) that degrades linear DNA.

**DNA nanoparticle formation**: To generate DNA nanoparticles, the DNA replication reaction with T7 DNA polymerase and primers (as above) was carried out overnight (for >12 h). Precipitates of DNA nanoparticles were collected by centrifugation at 10 000 *g* for 10 min, washed thrice with nuclease free water, and stored at 4 °C.

**Purification of Gp4 and Gp2.5**: T7 helicase Gp4 and single stranded DNA‐binding protein Gp2.5 were expressed as N‐terminal His_5_ tags from a pQE30 plasmid. Proteins were expressed from plasmids in *E. coli* M15 strain with pREP4, and induced with isopropyl β‐d‐1‐thiogalactopyranoside (IPTG, 1 mm) at 30 °C for 4 h. The cells were lysed by sonication. The His‐tagged proteins were enriched from the soluble fraction of cell lysate by using a Protino Ni‐TED column (Macherey–Nagel), purified with a gel filtration Superdex S200 column, and stored in a buffer containing potassium phosphate (pH 7.5, 20 mm), DTT (0.1 mm), EDTA (0.1 mm), and glycerol (20 %).

**Expression and purification of ParM**: The plasmid pET11a containing *parC*, the expression plasmids pET11a containing ParR, and ParM, containing five additional amino acids (GSKCK) at the C terminus to allow covalent attachment of fluorescent probes containing sulfhydryl‐reactive functional groups, were a kind gift from Paul Buske.^36^ BL21(DE3) cells were transformed with pET11a protein expression vectors under the control of a T7 promoter, grown to an OD_600_ of 1.0, and induced with lactose (2 %) overnight at 30 °C.

The cell pellet was resuspended in five volumes of lysis buffer containing Tris**⋅**HCl (pH 7.5, 30 mm), KCl (25 mm), MgCl_2_ (1 mm), DTT (2 mm), Triton‐X‐100 (0.1 %), phenylmethylsulfonyl fluoride (PMSF, 2 mm) and a small amount of DNase. Cells were lysed by sonication (6×5 min, 2 min cooling breaks in between). The cell lysate was clarified by centrifugation at 100 000 *g* and 4 °C for 1 h and subsequently subjected to an ammonium sulfate cut (0–40 %). It was centrifuged at 24 000 *g* for 30 min at 4 °C, the supernatant was discarded, and the pellet was resuspended in eight volumes of buffer A [Tris**⋅**HCl (pH 7.5, 30 mm), KCl (25 mm), MgCl_2_ (1 mm), DTT (2 mm)]. It was again clarified at 100 000 *g* at 4 °C for 1 h and then treated with ATP solution [ATP (10 mm), MgCl_2_ (1 mm), pH 7.5] for polymerization. These polymers were spun immediately at 100 000 *g* at 4 °C for 15 min. This procedure was performed twice, and the pellet was resuspended in 2 mL of buffer F [KCl (100 mm), Tris**⋅**HCl (pH 7.5, 30 mm), DTT (1 mm), MgCl_2_ (2 mm)]^36^ and gel‐filtered through a Superdex S200 column equilibrated in buffer F. Pure fractions were then determined by SDS‐PAGE, pooled, and frozen at −80 °C in glycerol (20 %).

**Expression and purification of ParR**: Proteins were expressed in BL21(DE3) strain with pET11a ParR. Cultures were grown in lysogeny broth (LB) and induced at OD_600_=1 with lactose (2 %) at 30 °C for 16 h.

The cell pellet was resuspended in three volumes of lysis buffer [2‐(morpholin‐4‐yl)ethanesulfonic acid (MES, pH 6.0, 50 mm), KCl (100 mm), EDTA (2 mm), glycerol (5 %), DTT (2 mm), PMSF (2 mm) and a small amount of DNase].

Cells were lysed by sonication (6×5 min, 2 min cooling breaks in between). The cell lysate was clarified by centrifugation at 100 000 *g* and 4 °C for 1 h and subsequently subjected to ammonium sulfate precipitation (0–50 %). It was centrifuged at 24 000 *g* for 30 min at 4 °C, the supernatant was discarded, and the pellet was resuspended in buffer 1 [50 mL; MES (pH 6.0, 25 mm), DTT (1 mm), EDTA (1 mm)]. It was again clarified at 100 000 *g* at 4 °C for 1 h and then rapidly loaded onto a MonoS column. Bound proteins were eluted with a gradient of NaCl (0–1 m). Enriched protein peak fractions analyzed on SDS‐PAGE were collected, concentrated to 2 mL in a YM‐10 centricon, and gel‐filtered with buffer R [KCl (300 mm), MES (pH 6.0, 30 mm), EDTA (1 mm), DTT (1 mm)] on an S75 column.^36^ Pure peak fractions were pooled after SDS‐PAGE analysis and stored in glycerol (20 %) at −80 °C.

All purified proteins were verified by MALDI MS.

**Labeling of ParM with fluorescent dyes**: DTT and glycerol were removed with a PD10 salt exchange column (by following the manufacturer's protocol) equilibrated in buffer F. For labeling, a commercially available kit was used (Alexa Fluor488 Protein Labeling Kit, Invitrogen, followed instructions according to the manufacturer's protocol). Average labeling efficiency was (90±10) %.

**Creation of** ***parC*** **with biotin/Cy3 moieties and DNA–bead coupling**: Primers that would generate a 356 bp stretch of DNA containing the *parC* sequence including a 5′‐biotin and a 3′‐cy3 moiety were designed. Spherical streptavidin‐coupled magnetic beads (Polysciences, 50 μL) were washed thrice with wash buffer [1.5 mL; Tris**⋅**HCl (pH 8.2, 10 mm), NaCl (1 m), EDTA (1 mm)] by using a magnetic separator. Beads were then resuspended in wash buffer (1.3 mL) plus Tween 20 (0.2 %). Biotinylated DNA (300 nm, 200 μL) was added to the tube, mixed, and incubated for 1 h at 4 °C. Beads were then washed with wash buffer (3×1.5 mL), followed by washing with buffer FE (2×1.5 mL; Tris**⋅**HCl (pH 7.0, 30 mm), KCl (100 mm), EDTA (1 mm). Beads were resuspended in FE (50 μL) and stored at 4 °C.

**Glass slide and coverslip preparation**: For passivation, commercially pre‐cleaned slides and coverslips were used. Slides and coverslips were again cleaned in a sonication bath with acetone, ethanol, isopropanol, and DI water (3 min each). The surface was dried under a nitrogen gas jet and subsequently plasma‐treated with oxygen plasma (45 s). Eventually, gas‐phase silanization was performed overnight by placing the slides in a desiccator. The silane (chlorotrimethylsilane, Sigma, approx. 200 μL) was placed in a small beaker in the desiccator and a mild vacuum was applied. The treated glass slides were stored in a dust‐free environment.

**R1 spindle assembly**: Centromeric DNA [14 pm DNA‐coated (*parC*) beads or enriched DNA nanoparticles] was combined with 30 % Alexa488‐labeled motor protein ParM (total concentration: 5 μm), adapter protein ParR (250 nm), methyl cellulose (0.4 %, 400 cP), DTT (5 mm), and bovine serum albumin (BSA, 15 mg mL^−1^) in buffer F. The reaction mixture was spotted on a glass slide, and the reaction was started with ATP (10 mm, one tenth of the reaction volume of 100 mm ATP). To reduce oxidation, reactions were sealed with nail polish after covering with a coverslip.

**Widefield fluorescence microscopy**: For widefield fluorescence microscopy an inverted epifluorescence microscope (Nikon Eclipse Ti‐U, Nikon Instrument, Japan) was used at 488 nm, with a 20× or 40× objective and a Zyla 4.2 Plus sCMOS camera (Andor Technology, Ltd, UK). The microscope was equipped with 525/50 nm or 647/57 nm mounted emission filters, respectively.

**Transmission electron microscopy of R1 spindles**: Samples were prepared as in the case of in vitro spindle assembly but without BSA. Polymerization was induced by addition of nucleotides, and samples (10 μL) were applied to carbon‐coated and glow‐discharged grids. These were subsequently stained with aqueous uranyl acetate (2 %). After rinsing by blotting with water twice and evaporation of the water, samples were visualized with a Tecnai T20 electron microscope at 200 kV.

**Production of biomimetic microcompartments and encapsulation of protein systems**: Water‐in‐oil droplets were prepared by use of a solution (2 %, *v*/*v*) of PFPE‐PEG‐PFPE surfactant “E2K0660”^61^ in HFE‐7500 fluorinated oil (from 3M). The surfactant was sourced from RAN Biotechnologies (http://www.ranbiotechnologies.com). Droplets were produced by mixing of surfactant‐stabilized hydrophobic phase (1.8 % surfactant and HFE‐7500 oil) with aqueous phase by vortexing. For encapsulation experiments the aqueous phase was the solution containing the appropriate protein systems (e.g. segregation machinery, oxygen‐scavenging system, ATP regeneration). The droplets were subsequently trapped in glass capillaries of 50 μm inner diameter (microcapillary tube, Sigma).

Microfluidic devices were constructed by standard photolithography techniques.^62^, ^63^, ^64^ A SU‐8 master was prepared on a silicon wafer, cast with freshly mixed poly(dimethylsiloxane) (PDMS; silicone elastomer kit SYLGARD 184, 1:10 crosslinker to base ratio, Dow Corning, USA) and polymerized overnight at 65 °C. After oxygen plasma treatment, microfluidic devices were bound on a glass slide. Lipid‐bilayer PDMS microcompartments were prepared as previously described.^65^

**O_2_‐scavenging system**: For the oxygen‐scavenging system, pyranose oxidase and catalase were purchased from Sigma. Final assay concentrations were 3.7 U mL^−1^ of pyranose oxidase and 90 U mL^−1^ catalase. Stocks (100×) of pyranose oxidase (38 mg mL^−1^) and of catalase (2 mg mL^−1^) were prepared by dissolving in suitable volumes of buffer F. The solution was filtered with use of centrifuge filters (0.22 μm). Aliquots (10 μL) were flash‐frozen in liquid nitrogen and stored at −80 °C. For use, equal volumes of both were mixed to provide a 50× solution. d‐Glucose needs to be added in a final concentration of 0.8 %. A 50× stock can be prepared with 40 % glucose. This was subsequently filtered for sterility and stored at −80 °C.

**ATP‐regeneration system**: The ATP‐regeneration system used was based on creatine kinase and creatine phosphate as substrate. To regenerate 0.1 mm ATP in 200 μL solution, creatine phosphate (20 mm) was added to creatine kinase (0.1 mg mL^−1^).

**Image processing and measurement of length and quantity distributions**: Microscopy images were processed with NIH Fiji ImageJ. Contrast or brightness adjustments were applied uniformly to the entire image field. The software was also used to determine spindle and aster length, as well as quantity‐of‐asters‐per‐bead distributions.

## Conflict of interest

*The authors declare no conflict of interest*.

## Supporting information

As a service to our authors and readers, this journal provides supporting information supplied by the authors. Such materials are peer reviewed and may be re‐organized for online delivery, but are not copy‐edited or typeset. Technical support issues arising from supporting information (other than missing files) should be addressed to the authors.

Supplementary

Supplementary

Supplementary

Supplementary

Supplementary

Supplementary

Supplementary

Supplementary

Supplementary
