# Supplementary material for: Reconstitution and Coupling of DNA Replication and Segregation in a Biomimetic System
Source: Chembiochem. 2019 Aug 28;20(20):2633–42. doi: 10.1002/cbic.201900299 (PMC6899551; doi:10.1002/cbic.201900299)
Supplement: Supplementary file 1 — Supplementary [file CBIC-20-2633-s001.pdf]

CHEMBIOCHEM

Supporting Information

Reconstitution and Coupling of DNA Replication and Segregation in a Biomimetic System

Daniel Hürtgen,*[a] Judita Mascarenhas,[a] Michael Heymann,[b] Seán M. Murray,[a] Petra Schwille,[b] and Victor Sourjik*[a]

cbic_201900299_sm_miscellaneous_information.pdf
cbic_201900299_sm_Movie-S1.avi
cbic_201900299_sm_Movie-S2.avi
cbic_201900299_sm_Movie-S3_scale.avi
cbic_201900299_sm_Movie-S4_scale.avi
cbic_201900299_sm_Movie-S5_scale.avi
cbic_201900299_sm_Movie-S6.avi
cbic_201900299_sm_Movie-S7.avi
cbic_201900299_sm_Movie-S8.avi

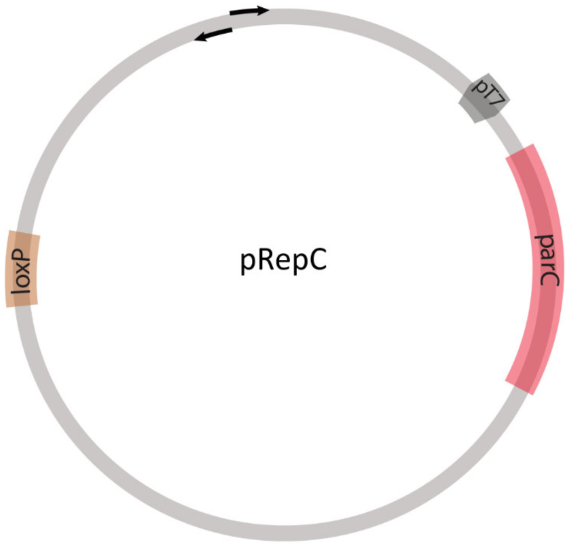

Figure S1: Schematic representation of the template plasmid pRepC used for T7-based replication. The total length of pRepC is 3.8 kb. Individual elements are not drawn to scale. Arrows depict annealing sites of specific replication primers.

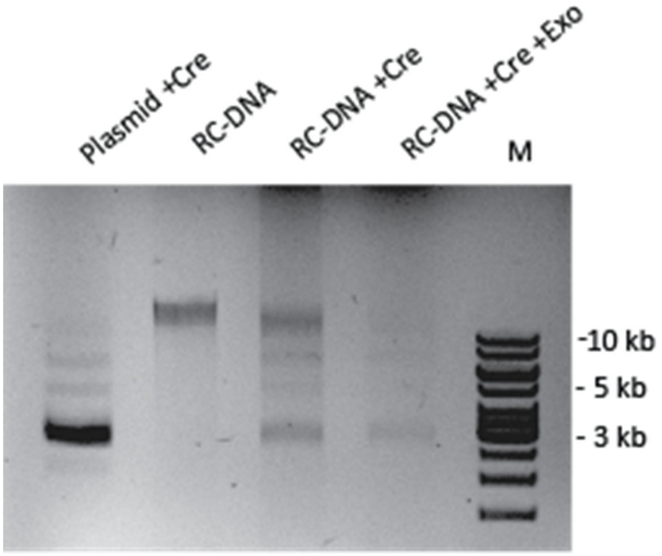

Figure S2: Circularization of replicated DNA using Cre recombination. Lane 1: plasmid (pRepC) treated with Cre recombinase; lane 2: product of rolling circle replication (RC-DNA); lane 3: RC-DNA treated with Cre recombinase; lane 4: reaction product as in lane 3 but additionally treated with exonuclease V(RecBCD) that enzymatically removes linear fragments.

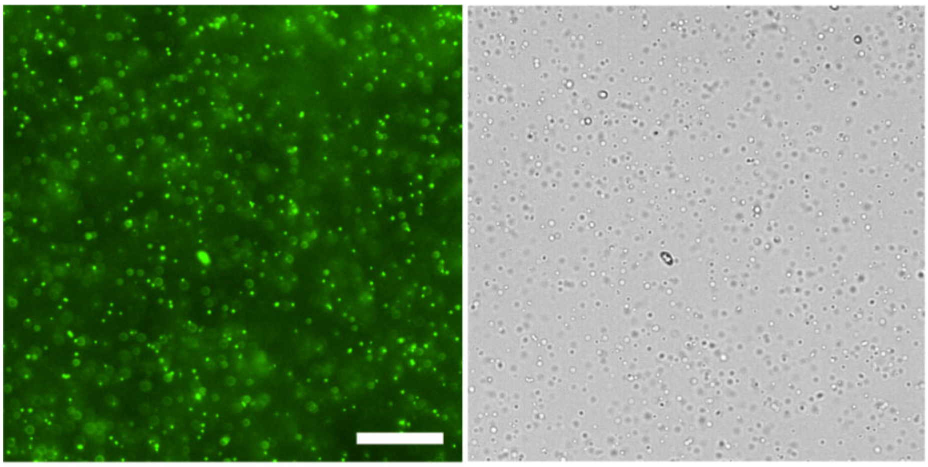

Figure S3: Staining of nanoparticles with DNA-binding dye. Nanoparticles formed upon prolonged replication as in Fig. 1C were stained with PicoGreen. Scale bar: 40 µm.

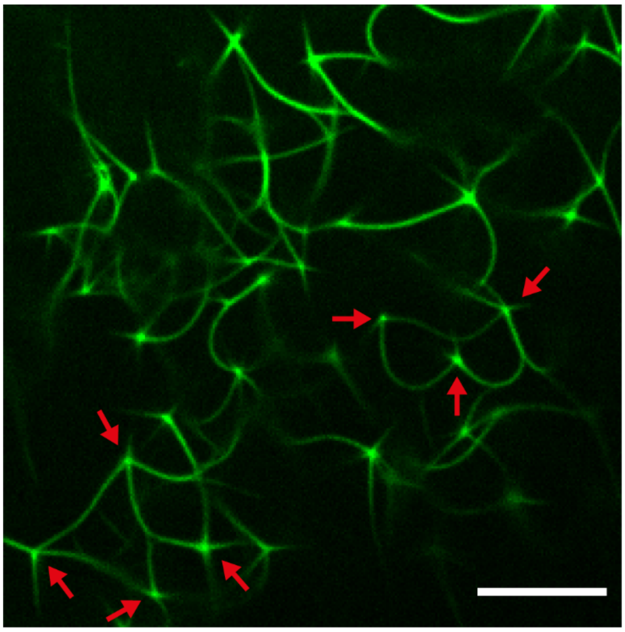

Figure S4: Formation of multipolar spindles by the ParMRC system. Multipolar spindles connecting several beads are indicated by red arrows. Scale bar: 10 µm.

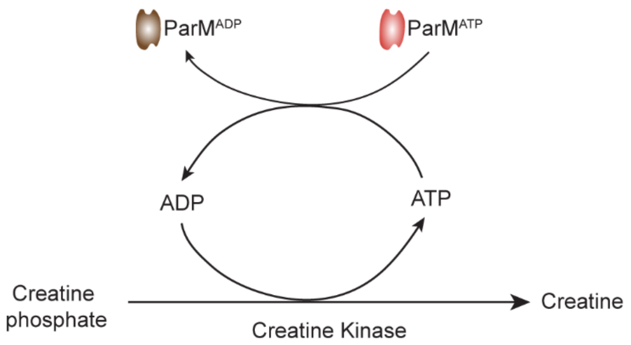

Figure S5: ATP regeneration system used in this study. Creatine kinase transfers one phosphate group from creatine phosphate to ADP in order to regenerate ATP.

Supplementary Movies

Movie S1: Bipolar Segregation. Movie of two artificial beads being segregated by a bipolar ParM spindle. Time lapse of the same reaction as in Figure 2F; 20 s/frame.

Movie S2: Multipolar Segregation. Movie of multiple artificial beads being segregated by multipolar ParM spindles. Time lapse of the same reaction as in Figure S2; 20 s/frame. Conditions as in Figure 2F.

Movie S3: Pendeling. Movie of pendeling behavior of artificial beads. Conditions as in Figure 2F. ; 20 s/frame.

Movie S4: Pendeling B. Movie of pendeling behavior of artificial beads. Conditions as in Figure 2F. ; 20 s/frame.

Movie S5: Bead Segregation in Microfluidic Channel**.** Movies of artificial bead-segregation. Conditions as in Figure 2F, but trapped within a BSA-coated microfluidic PDMS channel. Imaged is the Alexa-488 ParM channel. 20 s/frame.

Movie S6: Nanoparticle Segregation. Time lapse of the same reaction as in Figure 6C. One larger nanoparticle is sheared, while smaller fragments are being pushed apart by ParM filaments. Imaged is the Alexa-488 ParM channel. Frames are 20 s apart.

Movie S7: Nanoparticle Segregation B. Movies of nanoparticle segregataion. Imaged is the Alexa-488 ParM channel. Frames are 20 s apart.

Movie S8: Nanoparticle Segregation C. Movies of nanoparticle segregataion. Imaged is the Alexa-488 ParM channel. Frames are 20 s apart.
